# Changes in the profiles of smokers seeking cessation treatment and in its effectiveness in Galicia (Spain) 2001–10

**DOI:** 10.1186/1471-2458-14-613

**Published:** 2014-06-17

**Authors:** Elisardo Becoña, Ana López-Durán, Elena Fernández del Río, Úrsula Martínez

**Affiliations:** 1 Smoking Cessation Unit, Department of Clinical Psychology and Psychobiology, Faculty of Psychology, University of Santiago de Compostela, Santiago de Compostela, Spain; 2 Department of Psychology and Sociology, University of Zaragoza, Zaragoza, Spain

**Keywords:** Smoking, Profile, Cessation, Treatment

## Abstract

**Background:**

In recent years, the prevalence of daily smokers has decreased in all developed countries due to a great variety of factors. Despite this decrease, the effectiveness of clinical treatments has decreased and several studies report a change in smokers’ characteristics. The purpose of the present study is to analyze the changes in the characteristics of Spanish smokers who seek smoking cessation treatment between 2001 and 2010 and the changes in the effectiveness of such treatment.

**Methods:**

The sample was made up of 870 smokers who sought psychological treatment for giving up smoking at the Smoking Cessation Unit in the Faculty of Psychology of the University of Santiago de Compostela (Spain) during the period 2001 to 2010.

**Results:**

Smokers in the 2006–2010 group, compared to those in the 2001–2005 group, were older, smoked fewer cigarettes per day and of a brand with fewer mg/nicotine, had been smoking longer, were less motivated to give up smoking, and had more antecedents of depression. Quit rates were validated by testing smokers' carbon monoxide (CO) levels.

Percentages of abstinence were higher in the 2001–2005 group than in the 2006–2010 group (58.7% vs. 52.15 at the end of treatment, p = 0.05); 30.8% vs. 24.2% at 6 months follow-up, p = 0.031; 27.5% vs. 22% at 12 months follow-up, p = 0.059). Although abstinence decreased more than 5% in the 2006–2010 group there were no differences between the two groups in nicotine dependence. Those participants who did not assist to the follow-up were considered smokers at pretreatment level.

**Conclusions:**

In Spain there has been a qualitative change in the profile of the smokers seeking smoking cessation treatment. Treatment effectiveness has decreased, and the variables predicting intervention outcome have changed.

## Background

In recent years, the prevalence of daily smokers has decreased in all developed countries [1,2]. In Spain, according to the Spanish National Health Survey (*Encuesta Nacional de Salud*), 31.7% of people of 18 years or older smoked every day in the year 2001, whilst this percentage had fallen to 23.9% in 2012. However, the percentage of occasional smokers has remained stable in the last decade [3]. More specifically, according to a study conducted in Galicia, Spain, in 2005 [4], 25% of the population between 16 and 74 smoked daily, with a slightly higher prevalence of smoking in men than in women.

This significant decrease in the number of smokers may be due to a range of factors. Irvin, Hendricks and Brandon [5] consider that the main factors contributing to a 25% fall in smoking in the USA between 1983 and 1998 were: increased concern about the negative health consequences of smoking, the growth in limitations on smoking in public places, and increased availability of treatments for giving up smoking. In the same line, Lando [6] suggests that smoking cessation is more frequent in those contexts in which there are strong restrictions on this activity, such as those in which the price of cigarettes has increased significantly, or where smoking has come to be considered non-normative (through specific legislation).

With the aim of reducing the prevalence of smoking in Spain, two legislative measures have been implemented relatively recently (*Ley 28/2005* and *Ley 42/2010*). Until that moment, despite of the fact that many studies conducted worldwide had demonstrated the risks that smoke exposure had in health, smoking was allowed in working and leisure places in Spain. Moreover, legislation had focused only on the incorporation of health warnings in tobacco related products. The entry in 2006 of the *Ley 28/2005* and in 2011 the *Ley 48/2010* about health measures against tobacco were based on various regulatory aspects and on the promotion of programs and services for tobacco treatment and prevention [7]. These laws introduced the ban of tobacco advertising and promotion, the reduction of tobacco retail outlets and smoking bans in enclosed public spaces and workplaces.

After the introduction of these laws a positive impact in Spanish people health was noted, as it significantly diminished smoking prevalence in adolescents; the number of cigarettes smoked was decreased in people who continued smoking; and the morbidity for acute myocardial infarction reduced, an important indicator of the mortality attributable to tobacco [8,9].

Catalina et al. [10] consider these measures to have contributed significantly to the reduction in the number of smokers and the prevalence of smoking. However, according to Nebot and Fernández [11] this decrease in the number of smokers – and even in the number of cigarettes smoked per day in those who continue smoking – are due to the falling trend in relation to this habit observed before the introduction of this legislation. In this vein, recent reviews [12] conclude that specific legal prohibition with regard to smoking in public places would have a greater effect on passive than on active smoking, even though we cannot rule out the influence of these types of measures in the reduction of smoking among the general population.

In other countries, where this type of legislative measure has been applied for longer, there has been a more detailed study of the changes in smokers’ characteristics and in the effectiveness of smoking cessation treatments. Thus, for example, Fagerström et al. [13] and Fagerström and Furberg [14] analyzed the relationship between the prevalence of smoking and nicotine dependence in a range of countries. They concluded that as the number of smokers decrease, the level of nicotine dependence among those who continue to smoke increases (the so-called “hardening hypothesis”). This concept of “hardening” is associated with a decrease in the number of attempts to give up and with smokers’ reduced capacity or maintaining abstinence (due to their greater dependence). This results in a need to use different strategies in relation to the problem [15].

In this same way, Irvin and Brandon [16] point out that as tobacco use decreases in the general population, those who continue smoking are more difficult to treat, and this is reflected in a reduction in effectiveness of clinical treatments in recent years. Furthermore, these researchers report a change in smokers’ characteristics, greater use of other substances, higher levels of psychopathology, lower educational levels, lower social status and greater nicotine dependence.

The relation between the presence of psychopathology and the effectiveness of smoking cessation treatment has been widely studied. Piper et al. [17] and Schroeder and Morris [18], for example, conclude that people with problems of depression have more difficulty giving up smoking. In line with this, the presence of psychopathology should be considered when planning smoking cessation treatment. A possible explanation for the poorer outcomes in smokers with psychopathological problems, according to de Leon, Becoña, Gurpegui, González-Pinto and Díaz [19], is that these people would be more nicotine-dependent, this being one of the variables most closely related to poorer treatment results [20]. However, this view has been the object of criticism. For example, Shiffman, Brockwell, Pillitteri and Gitchell [21] hold that the relation between nicotine dependence and abstinence takes the form of a U. According to these authors, percentages of abstinence tend to be higher in less dependent smokers, but also in those with the greatest levels of dependence, to the extent that in some cases it is the latter who show the highest rates of treatment success. Smokers with moderate dependence, on the other hand, tend to obtain poorer results in smoking cessation treatments. A possible explanation for this is that those with most dependence have greater motivation for stopping smoking because they are subject to more serious health problems caused by their smoking [22], and health campaigns tend to be more especially focused on them [15].

Specifically, in our own context, we have perceived a significant increase in the number of people with antecedents of depression (having current or past major depressive episode) seeking smoking cessation treatment [23] over the last decade. In this line, Schroeder and Morris [18] point out that, although this type of population apparently responds to smoking cessation treatments in the same way as the general population, they tend to present higher nicotine dependence, so that they require more intensive interventions. So, these people with antecedents of depression may start experiencing such problems again on giving up smoking [24].

The aim of the present study was to analyze the changes in the characteristics of Spanish smokers who seek psychological treatment for smoking cessation in the period 2001 to 2010 and to examine changes in the effectiveness of such treatment.

## Methods

### Participants

The total sample was made up of people seeking psychological treatment for giving up smoking at the Smoking Cessation Unit in the Faculty of Psychology of the University of Santiago de Compostela (Spain) during the period 2001 to 2010 (N = 870; 43.2% men and 56.8% women; mean age = 40.64, *SD* = 10.69).

The smokers were selected according to the following inclusion criteria: age 18 or over, voluntary participation in the treatment programme, smoking a minimum of 10 cigarettes per day before the beginning of the treatment, and filling out all the questionnaires in the pretreatment assessment. Exclusion criteria were as follows: diagnostic of a severe mental disorder (such as bipolar disorder, psychotic disorder), concurrent cocaine or heroin dependence, having received the same treatment programme or another effective treatment for smoking cessation (nicotine gum, nicotine patches, bupropion or varenicline) over the last year, presenting a life-threatening pathology (e.g., recent acute myocardial infarction, pneumothorax or Chronic Obstructive Pulmonary Disease) that would require immediate treatment, and failing to attend the first session of the group treatment.

With the aim of comparing the evolution of smokers seeking treatment for giving up smoking, we compared two subsamples who received the treatment in the periods 2001–2005 (n = 465) and 2006–2010 (n = 405), respectively.

### Measures

Before starting the psychological treatment, all the smokers filled out a questionnaire [25] designed to gather information on both sociodemographic characteristics (e.g., gender, age) and characteristics related to smoking (e.g., number of cigarettes smoked per day, brand of cigarettes). This questionnaire also assesses the smoker’s stage of change at the time of seeking treatment, according to Prochaska and Diclemente’s [26] Transtheoretical Model of Change. This allowed participants to be categorized as being in the *Precontemplation*, *Contemplation* or *Preparation* stages of change prior to beginning the smoking cessation treatment.

Likewise, nicotine dependence was assessed by means of the Fagerström Test for Nicotine Dependence (FTND), using a cut-off point of 6 or more for this variable [14].

As regards the assessment of depressive symptomatology, we administered the MDE (Major Depressive Episode Screener) [27]. This is an instrument for detecting a major depressive episode in the past and/or in the last 15 days, based on the DSM-IV diagnostic criteria. During the interview, the smoker was also asked whether he or she had received treatment for depression in the past and/or recently.

Moreover, follow-up sessions were held at 6 and 12 months. During each follow-up session, questions were asked concerning the status of the individual (smoker/non-smoker) in that moment. The self-report abstinence in all time periods was corroborated through the measurement of expired breath carbon monoxide (CO). For this purpose we used the Micro IV Smokerlyzer (Bedfont Technical Instruments Ltd, Sittingbourne, Kent, UK). This device takes samples of expired breath via a sensor, indicating in particles per million (ppm) the participant’s carbon monoxide in expired air (CO) level.

### Procedure

The instruments described in the previous section were administered in the initial assessment. All the smokers gave written informed consent to take part in the study, and the research was authorized by the Bioethics Committee of the University of Santiago de Compostela.

The psychological treatment applied (Programme for giving up smoking, by Becoña [28,29]) is a cognitive-behavioral treatment with 6 sessions, and it is applied in a group format. Pharmacotherapy is not used. It comprises the following components: treatment contract, self-report and graphic representation of cigarette use, information about tobacco, stimulus control, activities for avoiding nicotine withdrawal syndrome, and physiological feedback on cigarette use via the measurement of CO in each session. Nicotine fading procedure is also used, changing cigarette brands each week decreasing progressively their intake of nicotine and tar. Finally, strategies for relapse prevention are used (assertiveness training, problem-solving strategies, change of erroneous beliefs, anxiety and anger management, physical exercise, weight control and self-reinforcement). Date of quitting is usually established between the fourth and fifth session. The treatment lasts 6 weeks (one session per week, of around one hour). Smokers are treated in small groups of 2–8 people. Assignment of the participants to groups took place according to their own timetable and availability; neither their sociodemographic nor their smoking characteristics are taken into account in this respect. Those applying the treatment were six psychologists with broad experience in the treatment of smokers. We found no differences in treatment outcomes as a function of the therapist assigned to each group.

Once the treatment was over, the research team carried out face-to-face follow-ups with the participants at 6 and 12 months. Both at the end of the smoking cessation treatment and in the follow-up sessions we adopted the criteria proposed by West, Hajek, Stead and Stapleton [30] for point prevalence abstinence at the end of the treatment (not having smoked in the last 24 hours, CO < 10 ppm) and for continued abstinence at 6 and 12 months (not having smoked, not even a puff, since the end of the treatment programme, CO < 10 ppm).

In those cases in which it was not possible to locate the participants for the follow-ups, they were considered smokers at the same level (number in cigarettes and milligrammes of nicotine) as in the pretreatment assessment.

### Statistical analysis

In order to determine the characteristics of the sample, we carried out statistical analyses of a descriptive nature. Likewise, for comparing the individuals treated in the two periods adopted as references (2001–2005 and 2006–2010), we used Pearson’s *χ*^2^ statistic for the categorical variables and the Student *t*-test for the categorical with numerical variables. In cases in which significant results were found, we included the corresponding effect size (Cramer’s V and Cohen’s d, respectively).

We also carried out a binary logistic regression analysis in each of the two groups of smokers to predict the key variables in the treatment outcomes over the course of 10 years. In the selection of the variables to form part of the logistic regression model we took as a reference a significance level of p < .05 in the univariate analysis. All the statistical analyses in this study were carried out with the IBM SPSS Statistics 20 package.

## Results

### Sociodemographic, smoking and psychopathological characteristics

We compared the two groups of smokers attending treatment over a 10-year period (Group 2001–2005 and Group 2006–2010). The results reveal that smokers who received treatment between 2006 and 2010 are older, smoked fewer cigarettes/day of a brand with lower nicotine content, have been smoking for less time (years) and were less motivated to give up than those smokers who attended treatment programmes between 2001 and 2005. We found no significant differences between the two groups as regards nicotine dependence. As far as psychological characteristics are concerned, in the 2006–2010 group there was a higher percentage of smokers who had experienced a major depressive episode at some time in their life and who had received treatment for depression in the past and at the time of seeking treatment for giving up smoking (see Table 1).

**Table 1:** Selected characteristics of smokers treated, by period

	**Group 2001**–**2005 (N = 465)**	**Group 2006**–**2010 (N = 405)**	***χ***^**2**^**/t**	**Cramer’s V/Cohen’s d**
**Sociodemographic characteristics**				
Gender (male)	43.0	43.5	0.02	
Age (41 or over)	45.6	54.8	7.37**	0.09
Mean age (SD)	39.1 (10.5)	42.4 (10.6)	-4.60***	0.16
Marital status (married)	46.9	50.1	7.24	
**Characteristics related to smoking**				
N° of cigarettes/day (25 or more)	47.1	35.6	11.86**	0.12
Mean cigarettes/day (SD)	25.9 (10.9)	23.1 (9.3)	4.20***	0.14
Milligrams of nicotine (0.8 mg or more)	88.2	81.0	8.68**	0.10
Mean milligrams of nicotine (SD)	0.8 (0.1)	0.7 (0.1)	6.49***	0.23
Years smoking (20 years or more)	59.4	67.2	5.66*	0.08
Mean years smoking (SD)	21.6 (10.1)	24.4 (10.6)	-3.96***	0.13
Has tried to give up smoking in the last year	44.1	38.8	2.52	
Stage of change (Preparation)	40.2	32.1	9.22*	0.10
Nicotine dependence (FTND ≥ 6)	49.0	46.9	0.39	
Mean nicotine dependence (SD)	5.24 (2.36)	5.24 (2.23)	0.01	
**Depression**				
MDE (past)	34.2	45.9	12.45***	0.12
MDE (current)	8.2	8.6	0.06	
Depressive disorder (past)	27.1	37.0	9.87**	0.11
Depressive disorder (current)	9.5	14.1	4.49*	0.07

Note: Means (SD) presented for continuous variables and percentages presented for numerical variables.

MDE = Major Depressive Episode; FTND = Fagerström Test for Nicotine Dependence.

*p < .05;**p < .01;***p < .001.

### Evolution of the effectiveness of smoking cessation treatment

**Figure 1:**
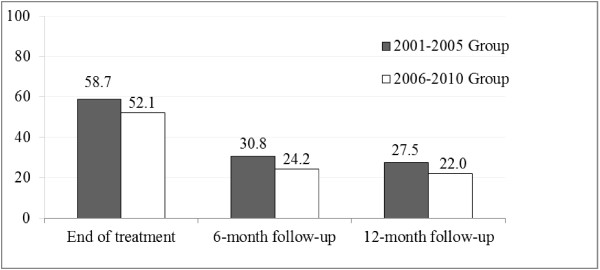
Abstinence at three stages of smoking treatment by period.

When comparing treatment outcomes over 10 years, we found a reduction in the effectiveness at the end of the treatment (58.7% vs. 52.1%; *χ*^2^ _(1)_ = 3.83; *p* = .05, Cramer’s V = 0.07, p = .05) and at the 6-month follow-up (30.8% vs. 24.2%; *χ*^2^ _(1)_ = 4.64, *p* = .03; Cramer’s V = 0.07, p = .03). At the 12-month follow-up, although it did not reach statistical significance (27.5% vs. 22%; *χ*^2^ _(1)_ = 3.56, *p* = .059), the percentage of abstainers within the group treated between 2001 and 2005 was also higher than that found in the 2006–2010 group (see Figure 1).

### Depression and abstinence over the course of the treatment

The presence of depressive problems was related to lower percentages of abstinence in the 2001–2005 group, both at the end of the treatment and at the 6- and 12-month follow-ups. In the 2006–2010 group there were only differences at the end of the treatment, since smokers with antecedents of depression obtained lower percentages of abstinence at the end of treatment (see Table 2).

**Table 2:** Percentages of abstinence at three stages of smoking treatment by depression history and period

	**Group 2001–2005 (N = 465)**			**Group 2006–2010 (N = 405)**		
		***χ***^**2**^	**Cramer’s V**		***χ***^**2**^	**Cramer’s V**
**End of the treatment**						
MDE (past)	47.2 (64.7)	13.27***	0.17	44.1 (58.9)	8.85**	0.15
MDE (current)	31.6 (61.1)	12.56***	0.16	40.0 (53.2)	2.25	
Depressive disorder (past)	46.0 (63.4)	11.45**	0.16	44.0 (56.9)	6.26*	0.12
Depressive disorder (current)	31.8 (61.5)	14.50***	0.18	50.9 (52.3)	0.04	
**6-month follow-up**						
MDE (past)	25.2 (33.7)	3.55		22.0 (26.0)	0.87	
MDE (current)	13.2 (32.3)	6.02*	0.11	28.6 (23.8)	0.40	
Depressive disorder (past)	25.4 (32.7)	2.33		19.3 (27.1)	3.07	
Depressive disorder (current)	6.8 (33.3)	13.07***	0.17	17.5 (25.3)	1.60	
**12-month follow-up**						
MDE (past)	20.1 (31.4)	6.63*	0.12	19.9 (23.7)	0.87	
MDE (current)	7.9 (29.3)	7.99**	0.13	17.1 (22.4)	0.52	
Depressive disorder (past)	23.0 (29.2)	1.76		18.0 (24.3)	2.19	
Depressive disorder (current)	9.1 (29.5)	8.28**	0.13	15.8 (23.0)	1.48	

Note: The percentage of abstinent individuals without antecedents of depression is shown in brackets.

MDE = Major Depressive Episode.

*p < .05;**p < .01;***p < .001.

### Variables that predict the success of the smoking cessation treatment

We conducted a logistic regression analysis separately for the two groups and we found that the factors associated with treatment success (i.e., abstinence) varied according to the time point considered.

In the group of smokers treated between 2001 and 2005, the variables significantly associated with abstinence at the end of the treatment were “being at the *Preparation* stage of change” (OR = 1.79) and “smoking fewer than 25 cigarettes/day” (OR = 1.88). In contrast, “having experienced a major depressive episode in the past” (OR = 0.59) and “receiving treatment for depression at the time of giving up smoking” (OR = 0.46) reduced the likelihood of attaining abstinence. As regards the predictor variables in the follow-ups, those participants with lower cigarette consumption pretreatment, and who were not receiving treatment for depression at that time were less likely to be abstinent in the medium term. Specifically, we found that at 6 months “smoking fewer than 25 cigarettes/day” (OR = 1.59) and “being in treatment for depression at the time of seeking treatment” (OR = 0.16) continued to be predictors of abstinence. At the 12-month follow-up, “being at the *Preparation* stage of change” (OR = 2.07) significantly increased the likelihood of remaining abstinent, whilst “being in treatment for depression at the time of seeking treatment” (OR = 0.24) reduced such likelihood (see Table 3).

**Table 3:** Predictors of abstinence at three stages of smoking treatment, by period

	**B**^**a**^	**Wald**	**p value**	**OR**	**CI 95%**
**Group 2001-2005**					
**End of the treatment**					
Stage of change (Preparation)	0.581	8.017	0.005	1.789	1.196-2.675
N° cig./day (<25 cig./day)	0.631	10.048	0.002	1.879	1.272-2.774
MDE past (yes)	-0.521	5.488	0.019	0.594	0.384-0.918
Depressive disorder (current) (yes)	-0.785	4.371	0.037	0.456	0.218-0.952
Constant	0.060	0.124	0.725	1.062	
**6-month follow-up**					
N° cig./day (<25 cig./day)	0.465	4.972	0.026	1.592	1.058-2.395
Depressive disorder (current) (yes)	-1.822	8.942	0.003	0.162	0.049-0.534
Constant	-0.962	35.605	0.000	0.382	
**12-month follow-up**					
Stage of change (Preparation)	0.728	11.730	0.001	2.071	1.365-3.142
Depressive disorder (current) (yes)	-1.424	6.997	0.008	0.241	0.084-0.691
Constant	-1.195	64.924	0.000	0.303	
**Group 2006-2010**					
**End of the treatment**					
N° cig./day (<25 cig./day)	1.035	22.324	0.001	2.815	1.833-4.325
MDE (past) (yes)	-0.689	10.820	0.001	0.502	0.333-0.757
Constant	-0.265	1.906	0.167	0.767	
**6-month follow-up**					
N° cig./day (<25 cig./day)	0.614	5.596	0.018	1.848	1.111-3.075
Constant	-1.560	50.293	0.000	0.210	
**12-month follow-up**					
N° cig./day (<25 cig./day)	0.577	4.622	0.032	1.781	1.052-3.013
Constant	-1.660	53.275	0.000	0.190	

Note. CI = confidence interval; FTND = Fagerström Test for Nicotine Dependence; ND = nicotine dependence; OR = odds ratio; MDE = Major Depressive Episode. The category of reference is shown in brackets.

^a^The groups were coded in the model as Smokers = 0 and Abstainers = 1.

With regard to the group of smokers treated between 2006 and 2010, we also found that “smoking fewer than 25 cigarettes/day” was a predictor variable associated with abstinence both at the end of the treatment (OR = 2.81) and at the 6-month (OR = 1.85) and 12-month follow-ups (OR = 1.78). On the other hand, “having experienced a major depressive episode in the past” was found to be associated only with a lower likelihood of giving up smoking at the end of the treatment (OR = 0.50) (Table 3).

## Discussion

The aim of the present study was to analyze the changes in the characteristics of Spanish smokers who received a psychological treatment for smoking cessation and the changes in the effectiveness of that treatment, between the years 2001 and 2010. This period was important, as it was when the Spanish government passed two relevant legislative measures in relation to the control of smoking.

The results reveal substantial differences between the smokers seeking treatment for giving up smoking at the Smoking Cessation Unit in the University of Santiago de Compostela (Spain) over a 10-year period (2001–2010). Those smokers who sought treatment in the period 2006–2010 were older, had been smoking for longer, smoked fewer cigarettes and of brands with lower nicotine and tar content, were less motivated to give up smoking, and had more depressive antecedents than those who applied for the same treatment in the period 2001 to 2005. The decrease in the number of cigarettes smoked daily in people who sought treatment is in line with the fall reported by Nebot and Fernández [11] in Spanish smokers. This decrease may be due, as suggested by Warner [31] and Yong, Borland, Thrasher and Thompson [32], to an increase of the social pressure (e.g., rise in the price of cigarettes, specific legislation for controlling consumption in certain spaces, etc.). It is not surprising that the number of cigarettes smoked per day has decreased if we take into account that it is forbidden to smoke in locations where it was permitted previously, such as the workplace or bars. As Villalbí points out [8], some indicators, as a decrease in tobacco sell figures, have been a clear reflection of the reduction of tobacco use in the Spanish population. However, although the number of cigarettes smoked appears to have declined, the decrease in the percentage of smokers is not that significant, which can also be due to the economical crises or to the increase of other types of tobacco, as can be roll-your-own cigarettes, that has also been indicated by previous studies [33].

On the other hand, the reduction in the level of motivation does not agree with what was proposed by Hughes [15], who points out that in the United States the remaining smokers are more motivated to give up smoking due to the growth of anti-smoking campaigns. According to a previous study conducted in Galicia, Spain [4], only 19% of the smokers were on the preparation stage, regardless of age or gender, and 44% of the total daily smokers had made some serious attempts to quit smoking. In contrast, the percentage of smokers of the clinical population in the preparation stage, despite having decreased in these ten years, they were significantly older. The lower motivation on seeking treatment found among the 2006–2010 group may be related to lower self-efficacy as regards the ability to give up smoking in a relatively short period of time (30 days). This low self-efficacy could, in turn, be related to the increased numbers of smokers with depressive problems in this period. According to previous studies [34], smokers with antecedents of depression would be highly motivated to give up smoking, but with low self-efficacy for achieving that goal. Thus, the fact that many of the smokers who come to treatment state that their intention to give up smoking is more in the medium term (6 months) than the short term (30 days) may be due to their low perceived self-efficacy, rather than to lower motivation to change. Therefore, it would be necessary to take into account both the different stages of the motivational process in which the smoker is situated at the time of the intervention [35] and his or her perceived self-efficacy.

As regards nicotine dependence, we found no differences between the two groups. Despite the prevalence of daily smokers in the general Spanish population has decreased (31.7% in 2001 and 23.9% in 2012), we have not found an increase in the level of nicotine dependence among those who continue smoking and seek treatment. This goes against the results reported in previous studies carried out in other countries [12,13,16]. So this does not confirm the “hardening hypothesis” among smokers seeking treatment. Recent studies conducted in smokers of the general population in Spain [36] or Italy [37] did not found support for this hypothesis either.

About the evolution of the effectiveness of the treatment for giving up smoking, the percentage of abstinence has decreased by more than 5%. Previous research in countries like the United States had already indicated such a trend [16]. In our case, the increased incidence of depression in the period 2006–2010 may explain the reduced effectiveness of the treatment in that period. As Piper et al. [17] and Schroeder and Morris [18] found, the presence of depression reduces the likelihood of success when a person tries to give up smoking. On the other hand, we cannot support the argument of de Leon et al. [19], since in our study the increase in psychopathological problems is not related to a rise in nicotine dependence that could explain the poorer treatment outcomes. Our results are in line with the view that smokers with moderate dependence have more problems for giving up smoking [21].

In relation to the variables that predict abstinence at the end of the treatment and in the follow-ups, we also found substantial differences. Whilst in the 2001–2005 group having antecedents of depression, a high rate of cigarettes smoked per day and low motivation for change predict lower likelihood of abstinence in any period, in the 2006–2010 group not having experienced a depressive episode is associated only with giving up smoking at the end of the treatment, and not with abstinence at the follow-ups. In this group, initial cigarette consumption is the most important variable for predicting abstinence. Therefore, having or not having depression is not anymore an important variable for predicting the success of smoking cessation treatment, at least in the long term. Perhaps due to the high percentage of smokers with this problem in the 2006–2010 group, depression has lost its predictive value, so that number of cigarettes smoked becomes the variable with the greatest weight for explaining the results in the follow-ups. Given the notable increase in numbers of smokers with depressive antecedents who wish to give up smoking, we might suggest, in line with Borrelli’s [38] proposal, the need to adapt smoking cessation treatment for this type of smoker, modifying aspects such as the mechanisms necessary for bringing about change, the way of intervention or the intensity of the programme, with the goal of improving treatment effectiveness.

Thus, the results obtained over these 10 years indicate that, in Spain, there has been a qualitative change in the profile of the smoker seeking psychological smoking cessation treatment (fewer cigarettes smoked, less motivation for change, and greater presence of depressive antecedents), but we have not found changes in nicotine dependence. Moreover, the effectiveness of smoking cessation treatment has decreased and there has been a significant change in the variables that predict intervention outcomes.

The present study has several limitations. First of all, the results obtained cannot be extrapolated to smokers in the general population, as smokers who seek specialized treatment for giving up the habit tend to be qualitatively different from those who do not [39,40]. Moreover, those studies that have reviewed the evolution of smokers’ characteristics have revealed different outcomes, as regards nicotine dependence, for example, according to whether the smokers in question are from the general population or clinical population [15]. Secondly, it might be advisable to assess the evolution of smokers’ dependence with other instruments, such as the NDSS-S (Nicotine Dependence Syndrome Scale-Short) [41] or the DSM criteria. Finally, we only took into account psychopathological antecedents related to depression. Various studies have stressed the need to take account of other types of disorder, such as anxiety disorders, mainly because of their high prevalence [42].

To summarize, over the last decade we have seen a significant fall in the prevalence of smokers in the general population. At the same time, however, as found in the present study, there has been a substantial change in the characteristics of smokers who seek specialized treatments for giving up smoking (and not always in the same line as in countries other than Spain) and a decrease in the effectiveness of such treatments. We consider it necessary to continue making progress toward improving interventions designed to address the serious health problem of smoking, adapting ourselves to the new profiles and demands of the population.

## Conclusions

There are important changes in the characteristics of smokers who seek smoking cessation treatment: 

1. Smokers in the 2006–2010 group are older, smoke fewer cigarettes/day and of a brand with fewer mg/nicotine, have been smoking for longer, are less motivated, and have more depressive antecedents than those in the 2001–2005 group.

2. In contrast to what has been found in other countries, we found no changes with regard to level of nicotine dependence between the two groups.

3. There has been a reduction in treatment effectiveness: percentage of abstinence decreased by more than 5% in the 2006–2010 group.

4. The variables predicting intervention outcome have also changed: in the 2001–2005 group those which are significant are number of cigarettes/day pretreatment, level of motivation and problems of depression. In the 2006–2010 group, depression is only significant at the end of the treatment, and the most significant variable is number of cigarettes/day pretreatment.

## Competing interests

The authors declare that they have no competing interests.

## Authors’ contributions

EB, ALD, EFR, UM designed the study and wrote the protocol. EB, ALD, EFR, UM obtained the data. EB, ALD, EFR, UM conducted the statistical analysis and contributed to the interpretation of the data. ALD, EFR drafted the manuscript. EB, UM provided feedback. All authors contributed to and have approved the final manuscript.

## Pre-publication history

The pre-publication history for this paper can be accessed here:

http://www.biomedcentral.com/1471-2458/14/613/prepub

## References

[B1] Bogdanovica I Godfrey F McNeill A Britton J Smoking prevalence in the European Union: a comparison of national and transnational prevalence survey methods and results Tob Control 2011 20 e4 20966129 10.1136/tc.2010.036103 PMC3003865

[B2] Mendez D Warner KE Adult cigarette smoking prevalence: declining as expected (not as desired) Am J Public Health 2004 94 251 252 14759934 10.2105/ajph.94.2.251 PMC1448235

[B3] Ministerio de Sanidad, Servicios Sociales e Igualdad Encuesta Nacional de Salud de España 2011–2012 [http://www.msssi.gob.es/estadEstudios/estadisticas/encuestaNacional/encuestaNac2011/PresentacionENSE2012.pdf. Accessed 27 July 2013]

[B4] de Galicia X O consumo de tabaco en Galicia, 2005 [Tobacco consumption in Galicia, 2005] Boletín Epidemiolóxico de Galicia 2006 XVIII 1 2

[B5] Irvin JE Hendricks PS Brandon TH The increasing recalcitrance of smokers in clinical trials II: pharmacotherapy trails Nicotine Tob Res 2003 5 27 35 12745504 10.1080/1462220031000070534

[B6] Lando HA Reflections on 30+ years of smoking cessation research: from the individual to the world Drug Alcohol Rev 2006 25 5 14 16492572 10.1080/09595230500459461

[B7] Córdoba R Villalbí JR Salvador-Llivina T López-García Aranda V El proceso en España de la adopción de una legislación eficaz para la prevención del tabaquismo [The process in Spain of adopting effective legislation to prevent smoking] Rev Esp Salud Publica 2006 80 631 645 17147303 10.1590/s1135-57272006000600004

[B8] Villalbí JR Valoración de la Ley 28/2005 de medidas sanitarias frente al tabaquismo [Assessment of the *Ley 28/2005* on health measures against smoking] Rev Esp Salud Publica 2009 83 805 820 20111829 10.1590/s1135-57272009000600005

[B9] Ministerio de Sanidad, Servicios Sociales e Igualdad, Secretaría General de Sanidad y Consumo Informe a las Cortes Generales de evaluación del impacto sobre la salud pública de la Ley 42/2010 [Report to the General Court of the assessment of the impact of the *Ley 42/2010* on public health] [http://www.msssi.gob.es/ciudadanos/proteccionSalud/tabaco/docs/Informe_Impacto_Salud_Ley_Tabaco.pdf]

[B10] Catalina C Sainz JC Quevedo L Cortés MV Pinto JA Gelpi JA Calvo E González A Prevalencia de consumo de tabaco en población trabajadora tras la entrada en vigor de la Ley 42/2010 [Prevalence of tobacco consumption among working population after the *Ley 42/2010*, Spain] Rev Esp Salud Publica 2012 86 177 188 22991060 10.1590/S1135-57272012000200006

[B11] Nebot M Fernández E Evaluación del impacto de la Ley de medidas sanitarias frente al tabaquismo [Assessment of the impact of the Law for health measures against smoking] 2009 Madrid: Ministerio de Sanidad y Política Social

[B12] Callinan JE Clarke A Doherty K Kelleher C Legislative smoking bans for reducing secondhand smoke exposure, smoking prevalence and tobacco consumption Cochrane Database Syst Rev 2010 doi:10.1002/14651858.CD005992.pub2 10.1002/14651858.CD005992.pub2 20393945

[B13] Fagerstrom KO Kunze M Schoberberger R Breslau N Hughes JR Hurt RD Puska P Ramstrom L Zatonski W Nicotine dependence versus smoking prevalence: comparison among countries and categories of smokers Tob Control 1996 5 52 56 8795860 10.1136/tc.5.1.52 PMC1759482

[B14] Fagerström KO Furberg H A comparison of the Fagerström test for nicotine dependence and smoking prevalence across countries Addiction 2008 103 841 845 18412764 10.1111/j.1360-0443.2008.02190.x PMC2914535

[B15] Hughes JR The hardening hypothesis: is the ability to quit decreasing due to increasing nicotine dependence? A review and commentary Drug Alcohol Depend 2011 117 111 117 21411244 10.1016/j.drugalcdep.2011.02.009 PMC3133840

[B16] Irvin JE Brandon TH The increasing recalcitrance of smokers in clinical trials Nicotine Tob Res 2000 2 79 84 11072444 10.1080/14622200050011330

[B17] Piper ME Smith SS Schlam TR Fleming MF Bittrich AA Brown JL Leitzke CJ Zehner ME Fiore MC Baker TB Psychiatric disorders in smokers seeking treatment for tobacco dependence: relations with tobacco dependence and cessation J Consult Clin Psychol 2010 78 13 23 20099946 10.1037/a0018065 PMC2813467

[B18] Schroeder SA Morris CD Confronting a neglected epidemic: tobacco cessation for persons with mental illness and substance abuse problems Annu Rev Public Health 2010 31 16.1 16.18 10.1146/annurev.publhealth.012809.103701 20001818

[B19] de Leon J Becoña E Gurpegui M González-Pinto A Díaz FJ The association between high nicotine dependence and severe illness may be consistent across countries J Clin Psychiatry 2002 63 812 816 12363123 10.4088/jcp.v63n0911

[B20] Fiore MC Jaén CR Baker TB Bailey WC Benowitz NL Curry SJ Dorfman SF Froelicher ES Goldstein MG Healton CG Henderson PN Heyman RB Koh HK Kottke TE Lando HA Mecklenburg RE Mermelstein RJ Mullen PD Orlenas CT Robinson L Stitzer ML Tommasello AC Villejo L Wewers ME Treating tobacco use and dependence: 2008 update 2008 Rockville, MD: U. S. Department of Health and Human Services, Public Health Service

[B21] Shiffman S Brockwell SE Pillitteri JL Gitchell JG Use of smoking-cessation treatments in the United States Am J Prev Med 2008 34 102 111 18201639 10.1016/j.amepre.2007.09.033

[B22] Bernstein SL Boudreaux ED Cabral L Cydulka RK Schwegman D Larkin GL Adams AL McCullough LB Rhodes KV Nicotine dependence, motivation to quit, and diagnosis among adult emergency department patients who smoke: a national survey Nicotine Tob Res 2008 10 1277 1282 18686174 10.1080/14622200802239272

[B23] Becoña E Míguez MC Group behaviour therapy for smoking cessation J Groups Addict Recover 2008 3 63 78

[B24] Hughes JR Depression during tobacco abstinence Nicotine Tob Res 2007 9 443 446 17454698 10.1080/14622200701243185

[B25] Becoña E Graña JL Evaluación de la conducta de fumar [Assessment of smoking behaviour] Conductas Adictivas: Teoría, evaluación y tratamiento [Addictive behaviours: Theory, assessment and treatment] 1994 Madrid: Debate 403 454

[B26] Prochaska JO DiClemente CC Stages and processes of self-change of smoking: toward an integrative model of change J Consult Clin Psychol 1983 51 390 395 6863699 10.1037//0022-006x.51.3.390

[B27] Muñoz R Preventing major depression by promoting emotion regulation: a conceptual framework and some practical tools Int J Mental Health Prom 1998 1 23 40

[B28] Becoña E Programa para dejar de fumar (Programme for giving up smoking) 1993 Santiago de Compostela: Servicio de Publicaciones de la Universidad de Santiago de Compostela

[B29] Becoña E Programa para Dejar de Fumar (Programme for giving up smoking) 2007 Vigo: Nova Galicia Edicións

[B30] West R Hajek P Stead L Stapleton J Outcome criteria in smoking cessation trials: proposal for a common standard Addiction 2005 100 299 303 15733243 10.1111/j.1360-0443.2004.00995.x

[B31] Warner K Tobacco control policy 2006 San Francisco: Jossey-Bass

[B32] Yong H Borland R Thrasher JF Thompson ME Stability of cigarette consumption over time among continuing smokers: a latent growth curve analysis Nicotine Tob Res 2012 14 531 539 22311963 10.1093/ntr/ntr242 PMC3337535

[B33] Yuong D Borland R Hammond D Cumming KM Devin E Yuong HH Prevalence attributes of roll-your-own smoker in the international tobacco control four country survey Tob Control 2006 15 Suppl III 17 23 10.1136/tc.2005.013268 PMC2593057 16754951

[B34] Haukkala A Uutela A Vartiainen E McAlister A Knekt P Depression and smoking cessation: the role of motivation and self-efficacy Addict Behav 2000 25 311 316 10795958 10.1016/s0306-4603(98)00125-7

[B35] Schlam TR Baker TB Interventions for tobacco smoking Annu Rev Clin Psychol 2013 9 675 702 23297788 10.1146/annurev-clinpsy-050212-185602 PMC5844577

[B36] Fu M Martínez-Sánchez JM Pérez-Ríos M López MJ Fernández E A comparison of the Fagerström test for nicotine dependence and smoking prevalence across countries: update data from Spain Addiction 2009 104 326 327 19149830 10.1111/j.1360-0443.2008.02485.x

[B37] Gallus S Pacifici R Colombo P La Vecchia C Garattini S Apolone G Zuccaro P Tobacco dependence in the general population in Italy Ann Oncol 2005 16 703 706 15817598 10.1093/annonc/mdi153

[B38] Borrelli B Smoking cessation: next steps for special populations research and innovative treatments J Consult Clin Psychol 2010 78 1 12 20099945 10.1037/a0018327

[B39] Hughes JR Giovino GA Klevens RM Fiore MC Assessing the generalizability of smoking studies Addiction 1997 92 469 472 9177068

[B40] Le Strat Y Rehm J Le Foll B How generalisable to community samples are clinical trial results for treatment of nicotine dependence: a comparison of common eligibility criteria with respondents of a large representative general population survey Tob Control 2011 20 338 343 21212379 10.1136/tc.2010.038703

[B41] Becoña E Fernández del Río E López A Míguez MC Castro J Nogueiras L Flórez G Alvarez S Vázquez D La Escala Breve de Evaluación del Síndrome de Dependencia de la Nicotina (NDSS-S) en fumadores españoles [The Short Nicotine Dependence Syndrome Scale (NDSS-S) in Spanish smokers] Psicothema 2011 23 126 132 21266153

[B42] Morissette SB Tull MT Gulliver SB Kamholz BW Zimering RT Anxiety, anxiety disorders, tobacco use, and nicotine: a critical review of interrelationships Psychol Bull 2007 133 245 272 17338599 10.1037/0033-2909.133.2.245

